# Solution Versus Suspension: How Does the Pharmaceutical Form of Insulin Shape the Mechanical Properties and Release Kinetics of a Carbopol^®^ Ultrez™ 10/Sodium Alginate Hybrid Hydrogel with a Molecular-Docking Rationale for Diffusion-Controlled Delivery

**DOI:** 10.3390/polym18202456

**Published:** 2026-10-09

**Authors:** Aneta Ostróżka-Cieślik, Sowmya Ramachandran, Wojciech Marczak

**Affiliations:** 1 Department of Pharmaceutical Technology, Faculty of Pharmaceutical Sciences in Sosnowiec, Medical University of Silesia, Jedności Street 8b, 41-200 Sosnowiec, Poland; 2 School of Pharmaceutical Sciences, Universiti Sains Malaysia, Gelugor 11800, Pulau Pinang, Malaysia; sowmyaar92@gmail.com; 3 Faculty of Science and Technology, Jan Długosz University, Al. Armii Krajowej 13/15, 42-200 Częstochowa, Poland; w.marczak@ujd.edu.pl

**Keywords:** insulin, hybrid hydrogel, Carbopol Ultrez 10, sodium alginate, topical delivery, release kinetics, Strat-M^®^ membrane, molecular docking

## Abstract

This study aimed to determine how insulin’s pharmaceutical form governs release from a topical matrix and to provide a molecular-level rationale for the observed behavior. A hybrid hydrogel of Carbopol^®^ Ultrez™ 10 and sodium alginate (1:2) was loaded with human insulin in two clinically distinct forms: a soluble solution (Actrapid^®^) and an isophane/NPH suspension (Insulatard^®^). Insulin release from the CU10:SA (1:2) hybrid hydrogel is continuous, without any sudden surge in release (the so-called *burst* effect), and follows diffusion according to Fick’s law. The soluble formulation released more insulin than the suspension, although the two profiles were similar in shape (*f*_1_ = 13.39; *f*_2_ = 97.54). The two CU10/SA insulin hydrogels were pseudoplastic fluids with pronounced shear-stress hysteresis and did not differ in hardness, cohesiveness, adhesiveness, or elasticity. Two rheological models, including the Carreau–Yasuda (CY) model and Santesarti’s shear rate-based model (SRB), described the recorded shear stress vs. shear rate curves fairly well. To explore this behavior at the molecular level, cavity-guided blind docking (CB-Dock2/AutoDock Vina) was performed on both the insulin monomer and the assembled zinc hexamer, using deprotonated oligomer models of the polymers. The alginate and Carbopol fragments converged on a single B-chain surface patch, where a carboxylate–Arg-B22 salt bridge supported by hydrogen bonding appeared to be the main anchoring motif, with weak docking scores (−4.5 to −4.9 kcal/mol on the insulin monomer) indicative of reversible physisorption rather than tight binding; the same patch was also engaged on the hexamer, which suggests that the proposed interaction motif does not depend on the insulin association state. These docking results are hypothesis-generating and complement, rather than prove, the experimental findings. The greater release of soluble insulin is attributed mainly to its pharmaceutical form, since the larger, less mobile protamine–zinc microcrystals of NPH insulin are likely to be more strongly entrapped in the matrix, rather than to differences in interaction chemistry. Together, the results link interaction chemistry, formulation state, and diffusion-controlled release.

## 1. Introduction

Pharmaceutical formulations containing peptides and proteins have grown rapidly. Synthetic peptides for clinical use accounted for over 11% of new chemical entities (NCEs) approved by the US Food and Drug Administration (FDA) between 2016 and 2024, and the number of registered peptide-based medicines continues to rise [1]. The main limitations of pharmaceutical peptides and proteins include poor stability due to proteolytic and chemical degradation, large molecular size, and hydrophilicity, hindering absorption across biological membranes and transdermal transport. Complex drug manufacturing processes (e.g., elevated temperatures and homogenization) can also damage therapeutic protein molecules [2]. A literature review indicates that hydrogels can serve as effective carriers for large, labile protein-peptide molecules for transdermal administration. Hydrogel matrices are produced under conditions that favor protein stability (room temperature, aqueous environment). The molecules are immobilized within a three-dimensional hydrogel network, preserving their bioactivity and 3D structure. As a result, they are less susceptible to proteolysis and aggregation [2]. They also provide a convenient dosage form and controlled release of the active pharmaceutical ingredient (API) via diffusion, swelling, erosion/degradation, or a combination of these mechanisms [2,3,4].

Hydrogels enhance the delivery of therapeutic proteins to affected sites, including chronic and hard-to-heal wounds. They offer many benefits, including tissue adhesion, hemostatic action, maintenance of a moist wound environment, removal of wound exudate and preservation of integrity in the presence of wound exudate, prevention of secondary infections, and self-healing. Because peptides and proteins have short half-lives, hydrogel matrices can extend their half-lives [5]. Preclinical and clinical studies indicate the therapeutic efficacy of insulin, a peptide hormone, in treating chronic wounds, including diabetic wounds and burns. Its biological activity is triggered by binding to a specific transmembrane receptor on the cell. Insulin interacts with receptors located on various skin cells, namely keratinocytes, fibroblasts, and endothelial cells [6,7,8,9]. Liu et al. [10] confirmed that topical insulin application to wounds in mice with experimental skin excision accelerates re-epithelialization and stimulates tissue healing.

Insulin binding to the insulin receptor (IR) activates the PI3K-Akt-Rac1 pathway and stimulates keratinocytes to produce integrin α3 and LN332. Akt1 activation increases vascular endothelial growth factor (VEGF) production in keratinocytes by phosphorylating 4E-BP1 [11]. Other authors [12] have suggested that insulin incorporated into a chitosan-based hydrogel matrix containing dipic acid dihydrazide (ADH) accelerated wound healing and alleviated peripheral neuropathy. The dressing shortened the inflammatory phase, stimulated granulation tissue formation, promoted collagen deposition, and accelerated re-epithelialization and the formation of new blood vessels (neovascularization). In a subsequent study [13], insulin incorporated into an alginate-diamine PEG-g-poly (PEGMA)-based hydrogel retained its structural stability and bioactivity. Studies conducted in a rat model of post-operative wounds with induced diabetes showed that the hormone plays a significant role in regulating the inflammatory phase and macrophage polarization. Insulin promoted the Akt/mTOR pathway, contributing to rapid wound closure (95% within 14 days). The hormone was released in a controlled manner (70% within 48 h) at physiological pH.

Dawoud et al. [14] conducted a clinical trial involving 15 patients with chronic wounds and used a chitosan hydrogel with insulin-loaded liposomes developed by their team. The authors witnessed a 16-fold improvement in wound healing rates compared with the control group. They also observed reduced erythema within the ulcer, with no symptoms of hypoglycemia. Furthermore, a systematic review and network meta-analysis by Ramirez-Garcia-Luna et al. [15] confirmed that topically applied insulin (as a spray, injection, or gel) promotes wound healing, with a limited risk of adverse effects. The authors suggested that the therapy is safe for both diabetic and non-diabetic patients and does not significantly change blood glucose levels. Hydrogel insulin carriers represent a promising therapeutic strategy for treating hard-to-heal wounds.

The current study aimed to carry out pre-formulation tests and to determine how the pharmaceutical form of insulin (solution vs. suspension) modifies the mechanical properties of the hydrogel matrix and the kinetics of hormone release, besides providing a molecular-level rationale for the observed release behavior by mapping insulin–polymer interactions in silico, with a view to optimizing the technology for preparing insulin hydrogels. Commercially available insulin preparations were proposed as a source of the active ingredient with a view to the potential preparation of insulin hydrogel formulations in a pharmacy setting.

This report presents the computational and mechanistic components of a study on a hybrid hydrogel intended for topical insulin delivery. The hydrogel is a physically blended matrix of Carbopol^®^ Ultrez™ 10 (CU10; a cross-linked poly (acrylic acid)) and sodium alginate (SA), formulated at a 1:2 (CU10:SA) ratio and loaded with insulin in two clinically distinct forms: a soluble formulation (Actrapid^®^) and an isophane (NPH) suspension (Insulatard^®^). The computational component aims not to replace the experimental findings but to provide a molecular-level rationale for the observed diffusion-controlled release behavior and the difference between the soluble and suspension formulations. To this end, the present study used molecular docking to characterize interactions between insulin and the two polymer components of the matrix.

## 2. Materials and Methods

### 2.1. Materials

Insulatard^®^ Penfill^®^ (100 IU/mL, suspension of human insulin, long-acting NPH isophane); excipients: zinc chloride, protamine sulfate, glycerol, metacresol, phenol, sodium hydrogen phosphate dihydrate, sodium hydroxide, hydrochloric acid (Novo Nordisk^®^ A/S, Novo Alle, Bagsværd, Denmark). Actrapid^®^ Penfill^®^ (100 IU/mL, solution of rapid-acting human insulin); excipients: zinc chloride, glycerol, metacresol, sodium hydroxide, hydrochloric acid (Novo Nordisk^®^ A/S, Novo Alle, Bagsværd, Denmark). Sodium alginate (W_w_ = 629 kDa) was sourced from Agnex Sp. z o.o., Białystok, Poland. Calcium chloride dihydrate (98.5% purity) was sourced from POCH S.A., Gliwice, Poland. Glycerol 86% was supplied by PPH Microfarm, Zabierzów, Poland. Carbopol^®^ Ultrez™ 10 (INCI: Carbomer) was supplied by Lubrizol Advanced Materials Inc., Cleveland, OH, USA. Triethanolamine (TEA) was supplied by Chempur, Piekary Śląskie, Poland. Phosphate-buffered saline (PBS) was sourced from Sigma-Aldrich (St. Louis, MO, USA). All substances used were of the highest purity grade. The Strat-M^®^ membrane was purchased from Merck Millipore (Burlington, MA, USA).

### 2.2. Hydrogel Formulation and Insulin Loading

A sodium alginate-based formulation was synthesized by dissolving 5.0% SA (*w*/*w*) in a mixture of 83.0% (*w*/*w*) PBS and 10.0% (*w*/*w*) glycerol. The gelation process was carried out by ionic cross-linking, adding 2.0 g of 0.5% calcium chloride (CaCl_2_) and stirring until a hydrogel formed. A preparation containing Carbopol^®^ Ultrez™ (CU10) was produced by gradually adding 1% (*w*/*w*) CU10 to a mixture of 93.0% (*w*/*w*) PBS and 6.0% (*w*/*w*) glycerol. Once hydration was complete, neutralization was carried out with triethanolamine (neutralisation was carried out by gradually adding TEA until a pH of 7.0 ± 0.1 was achieved, as monitored using a pH meter), and the mixture was continuously stirred until a three-dimensional (3-D)hydrogel structure formed. The resulting components were combined in a 1:2 ratio (CU10:SA). The hydrogel matrices were formulated using an HT-50DX mechanical stirrer (Witeg Labortechnik GmbH, Wertheim, Germany). The rotational speed was set to 1500 rpm. The preparations were left for 7 days until the system reached equilibrium, at 2–8 °C. Insulin was added manually at a rate of 1 mL per 2.5 g of hydrogel (dose of 28.57 IU/g) using two commercial preparations: Actrapid^®^ Penfill^®^ (soluble human insulin, 100 IU/mL, pH 7.12) and Insulatard^®^ Penfill^®^ (suspension of human isophane/NPH insulin, 100 IU/mL, pH 7.30). The formulations were stored between experiments in a refrigerator at 4 °C. Table 1 summarizes the physicochemical parameters of the finished insulin hydrogels. pH measurements were carried out using a SevenCompact™ S210 pH meter fitted with an InLaB^®^ Expert Pro-ISM electrode (Mettler-Toledo GmbH, Greifensee, Switzerland). The osmotic pressure of the preparations was determined using a Gonotec Osmomat 3000 Gonotec GmbH, Berlin, Germany. The mechanical stability of the hybrid hydrogels was tested by centrifugation (MPW-300 centrifuge, MPW Med. Instruments, Warsaw, Poland) at 4000 rpm for 30 min. Stability tests on the hydrogels were carried out in accordance with the guidelines on “Stability testing of biotechnology/biological products” as set out by the International Conference on Harmonisation (ICH), Q5C (storage temperatures of 25 ± 1 °C and 5 ± 3 °C). Monitoring was carried out based on visual assessment, pH, rheological properties, and hormone content. The samples remained stable over a 4-week storage period [16].

### 2.3. In Vitro Insulin Release Measurements

To assess pharmaceutical availability, a USP Apparatus 2 paddle-type apparatus (DT600 Erweka, Husenstamm, Germany) fitted with vertical Dissolution Enhancer Cell™ chambers (exposure area 3.80 cm^2^, Erweka, Husenstamm, Germany) was used. The diffusion chambers (*n* = 6) were prepared according to the manufacturer’s instructions, with a Strat-M^®^ membrane (a synthetic membrane that mimics the skin barrier) placed inside each chamber to separate the insulin hydrogel sample (1 g) from the PBS acceptor fluid (pH = 7.4). In each case, uniform contact between the preparation and the membrane was ensured. Each chamber was placed at the bottom of a 200 mL vessel. The test was conducted at 32 ± 1 °C (human skin temperature), with a 50 mL volume of acceptor fluid. The mini-paddle rotated at 100 rpm. Sink conditions were maintained throughout the test. Aliquots were withdrawn at defined time intervals over the study period (0–420 min in the present dataset). Insulin concentration in the acceptor fluid was determined by UV–Vis spectrophotometry at λ = 271 nm using a previously validated calibration curve with *R*^2^ = 0.999 [17,18,19,20]. Cumulative release was calculated with correction for the volume removed at each sampling point.

The Strat-M^®^ membrane can be used as a substitute for human skin in in vitro studies of the pharmaceutical availability of an active principal (comparable results to those obtained using human skin have been observed). The membrane contains a top layer corresponding to the epidermis. It also comprises a mixture of lipids corresponding to those found in the stratum corneum of human skin. Its advantage lies in the ability to conduct research without the use of animals, in accordance with the principles of animal welfare [18].

The research model used made it possible to assess the pharmaceutical availability of the active substance from the preparation under in vitro conditions, in accordance with pharmacopoeial guidelines on the quality assessment of preparations for topical application.

### 2.4. Computational Methodology (Molecular Docking)

#### 2.4.1. Receptor (Insulin) Preparation

The three-dimensional structure of human insulin was obtained from the Protein Data Bank (PDB ID: 1MSO; T6 human insulin, 1.0 Å resolution) [21]. The human insulin structure was specifically selected to align with the human insulin used in the formulations Actrapid^®^ and Insulatard^®^. Notably, it includes the human-specific threonine (Thr) at position B30. As a first, simplified model of the solvent-exposed insulin surface, the biological assembly was reduced to a single insulin monomer (one A-chain, 21 residues; one B-chain, 30 residues). Crystallographic water molecules and zinc ions were removed, and the disulfide-bonded monomer was retained. Hydrogen addition and partial-charge assignment were handled by the docking preparation pipeline (Section 2.4.3), with protonation appropriate to the formulation pH (~7.0–7.2).

To test the influence of insulin self-association, the biological hexamer (the 1MSO asymmetric-unit dimer expanded by the crystallographic three-fold to the physiological zinc hexamer; 12 chains) was also prepared as an alternative receptor, with waters and zinc removed as for the monomer, and both polymer fragments were re-docked under identical CB-Dock2/Vina settings.

Zinc was removed from both receptors for three reasons, even though the NPH suspension natively contains it. First, in the T6 hexamer each zinc ion sits on the three-fold axis inside the central channel, where it is coordinated by three His-B10 residues and three water molecules [21]. In the 1MSO structure, it lies more than 21 Å from the guanidinium group of any Arg-B22 and is therefore well away from the surface pocket that a diffusing polymer segment can reach. Second, CB-Dock2 runs AutoDock Vina 1.1.2 [22], whose scoring function has no term for zinc coordination, and zinc-specific parameters are available only in the separate AutoDock4Zn force field [23]. Retaining the ions would therefore introduce poorly described charges rather than make the model more physiological. Third, removal of bound ions and ordered water molecules is standard receptor preparation for blind docking, because atoms left in place would occupy or reshape the cavities identified in the detection step. The structural contribution of zinc is preserved, because the hexamer geometry is taken from the zinc-bound crystal structure. Its electrostatic contribution to the central channel is treated as outside the scope of the surface interaction studied here, and the single cavity located inside that channel is excluded from interpretation (see the Robustness to Insulin Assembly State Section).

#### 2.4.2. Ligand (Polymer Fragment) Preparation

Because full polymer networks cannot be realistically docked, each polymer was represented by a short, chemically faithful oligomer fragment:

Alginate was represented by a uronic-acid disaccharide (mannuronic/guluronic type; C_12_H_18_O_13_) bearing two carboxyl groups and a glycosidic linkage. During preparation, an initial database-derived model was found to contain spurious phosphorus atoms (a misannotated 3-D structure); this was corrected to a phosphorus-free uronic-acid disaccharide before docking.

Carbopol was represented by a poly(acrylic acid) trimer (three carboxyl groups; SMILES CC(C(=O)O)CC(C(=O)O)CC(C(=O)O)), which served as the primary model. A single acrylic-acid unit (the isolated repeat unit) was also docked for reference.

Ligand 3-D geometries were generated from SMILES within CB-Dock2. Critically, the carboxyl groups of both polymers were modeled explicitly in their deprotonated (carboxylate, –COO^−^) state, consistent with their ionization at the formulation pH (~7); this is the form that governs the electrostatic interaction with insulin, and the deprotonated ligands carried the correct formal charge in the docking input. Treating every carboxyl group as ionized is nevertheless a simplification, because neighboring carboxyl groups on poly(acrylic acid) are not all ionized at neutral pH, and this likely overstates the electrostatic contribution to the docking scores.

These fragments are not intended to reproduce the macroscopic diffusion barrier of the gel, which is determined by the cross-linked network and is characterized directly by the release and rheological experiments. Their role is narrower. Docking is used here to identify which chemical groups on each polymer contact insulin and where they do so. For a linear polyelectrolyte, the repeat unit sets the identity of those groups since a carboxylate on an alginate uronic residue, or on an acrylic-acid unit, presents the same local chemistry whether it sits on a short fragment or a long chain. A representative oligomer therefore captures the kind of contact that a diffusing segment makes at the insulin surface. Chain length mainly affects the score magnitude, which increases with oligomer size in docking studies of oligosaccharides binding to plasma proteins [24], and long, highly flexible chains are also poorly sampled as docking ligands. The scores therefore reflect interaction tendency at the repeat-unit level, not affinities of the whole network.

#### 2.4.3. Molecular Docking

Cavity-detection-guided blind docking was performed with the CB-Dock2 web server [22], which integrates automatic cavity detection (CurPocket) with AutoDock Vina [23,25]. Docking was performed with AutoDock Vina 1.1.2 via CB-Dock2 (accessed on 6 July 2026) using default exhaustiveness; nine poses per cavity were generated, and the lowest-energy pose was retained. For each ligand, the prepared insulin monomer was used as the receptor, the five top-ranked surface cavities were identified, and Vina docking was carried out within each cavity using automatically sized search boxes (edge lengths of 20 Å, 19 Å, and 15 Å for the alginate disaccharide, the Carbopol trimer, and the acrylic-acid unit, respectively). Identical receptor and server settings were used across ligands so that the alginate–Carbopol comparison depended only on the ligand.

#### 2.4.4. Interaction Analysis

For each ligand, the lowest-energy (most negative Vina score) pose was retained. Docking scores (kcal/mol), the insulin residues in contact with the ligand, and the nature of the interactions (hydrogen bonding, electrostatic/salt-bridge, and hydrophobic/van der Waals contacts) were analyzed within the CB-Dock2 3-D viewer. Because docking scores are size-dependent, ligand efficiency (docking score per non-hydrogen atom) was also considered.

### 2.5. Rheological Measurements

The rheological measurements were carried out using an RM 200 rotational rheometer fitted with a CP-1 PLUS heating system (both Lamy Rheology Instruments, Champagne-au-Mont-d’Or, France). An MK-CP 2445 measuring system was used in a plate-on-plate configuration (shear rate 7.0 s^−1^ to 100.0 s^−1^). Measurement accuracy was ±1%, and repeatability was ±0.2%. Rheological characterization was carried out at 25 °C ± 0.1 °C. Thixotropic analysis was carried out using the hysteresis loop method. The specimens were subjected to shear stress changes at a gradually increasing shear rate (not exceeding 100 s^−1^) and then at a decreasing shear rate. The analysis was carried out using Rheomatic-P software (Version: 2.1.0.4).

### 2.6. Texture Analysis

Texture analysis was carried out using a TX-700 texture analyzer (Lamy Rheology Instruments, Champagne au Mont d’Or, France), fitted with an 8 mm hemispherical probe. Two tests were carried out, namely CRT (Direct Compression/Relaxation/Tension) and TPA (Tension/Penetrometry), at a temperature of 25 °C ± 0.1 °C. The analysis was carried out using the RheoTex software for the TX-700, version TX-UK01/2019. The theoretical basis for the texture analysis is presented in a previous article [20].

### 2.7. Statistical Analysis and Model Fitting

Student’s *t*-test was used to assess the statistical significance (*p* < 0.05) of the data obtained. The two cumulative release profiles were compared by a paired two-tailed *t*-test on the matched sampling times, with the Wilcoxon signed-rank test as a distribution-free confirmation; the areas under the release curves (AUC_0–420_) were obtained by the trapezoidal rule. The calculations were performed using Statistica, version 13 (TIBCO Software, Inc., Palo Alto, CA, USA) [26].

Model equations were fitted to the empirical data using the non-linear fitting module of Statistica 13 [26]. The quasi-Newton algorithm was used to calculate the weighted least squares regression, with the reciprocal variances of the measurement results used as weights. For unweighted least-squares fitting, the Levenberg–Marquardt algorithm was applied. Statistically insignificant model coefficients were rejected based on *t*-test results.

Goodness of fit was assessed using the adjusted coefficient of determination $R_{\mathrm{adj}}^{2}$:

(1) $$R_{\mathrm{adj}}^{2}=1-\left(1-R^{2\;}\right)\frac{n-1}{n-p},$$

where *n* is the number of data points, and *p* is the number of parameters in the model.

Readers should be aware that $R_{\mathrm{adj}}^{2}$ is subject to well-founded criticism [27], and its application to models without adjustable intercept coefficients is questionable. To compare rheological curves, other criteria, such as the Akaike Information Criterion (*AIC*) [28] and the Model Selection Criterion (MSC), were also employed:

*AIC* = *n* ⋅ *ln*(*WSS*) + 2*p*, (2)

where *n* is the number of data points, *WSS* is the weighted sum of squares, and *p* is the number of parameters in the model:

(3) $$\mathrm{MSC}\;=\ln\left(\frac{\sum_{i=1}^{n}w_{i}\cdot {\left(y_{i_{\mathrm{obs}}}-\;{\bar{y}}_{\mathrm{obs}}\right)}^{2}}{\sum_{i=1}^{n}w_{i}\cdot {\left(y_{i_{\mathrm{obs}}}-y_{i_{\mathrm{pre}}}\right)}^{2}}\right)-\frac{2p}{n}\;,$$

where *w_i_* is the weighting factor, $y_{i_{\mathrm{obs}}}$ is the i-th observed *y* value, $y_{i_{\mathrm{pre}}}$ is the *i*-th predicted *y* value, $\bar{y}$*_obs_* is the mean of all observed *y*-data points, *p* is the number of parameters in the model, and *n* is the number of data points.

They can, however, be faulted for the same reasons as $R_{\mathrm{adj}}^{2}$. However, as long as $R_{\mathrm{adj}}^{2}$, AIC, or MSC are not used to compare a model with an adjustable intercept to one without such a coefficient, they can serve the purpose. Indeed, they are commonly used provisional measures of fit quality.

The similarity of release profiles was evaluated using the factors of difference (*f*_1_) and similarity (*f*_2_) in accordance with the recommendations of the FDA and the European Medicines Agency (EMA) [29,30]:

(4) $$f_{1}=\left[\frac{\sum \left|R_{t}-T_{t}\right|}{\sum R_{t}}\right]\cdot 100,$$

(5) $$f_{2}=50\cdot \log\left\{\left[1+\right.\frac{1}{n}\sum \left(R_{t}\right.-T_{t}{)}^{2}{]}^{-0.5}\cdot 100\right\},$$

where *n* is the number of time points, *R_t_* is the reference sample at time *t*, and *T_t_* is the test sample at time *t*.

## 3. Results and Discussion

### 3.1. Characteristics of the Hydrogels

The hydrogels were developed from two hydrophilic polymers, sodium alginate and Carbopol^®^ Ultrez™ 10, both widely used as excipients in drug formulation technology. The proposed polymer system combines the biocompatibility of SA with the mechanical strength of CU10, enabling formulations with optimal functional characteristics. Many researchers use this technology to improve the mechanical and physical properties of biodegradable hydrogel matrices [31,32].

The pH of the prepared hydrogels, as determined by the measurements carried out, was close to neutral at 7.02 and 7.16 (compared with Insulatard^®^ Penfill^®^ pH = 7.3 and Actrapid^®^ Penfill^®^ pH = 7.12), which ensured the insulin structural stability, bioactivity, and conditions conducive to cell proliferation (pH = 7.0–7.5) [33]. The viscosity results at a shear rate of 30 s^−1^ (Table 1) indicated that the CU10/SA–INS suspension (Insulatard^®^) hydrogel had a more compact structure (7.9 Pa·s vs. 7.0 Pa·s), which typically results in greater API retention within the matrix. And a slower rate of release [32]; this expectation is borne out by the release data (Section 3.2) and by the flow parameters in Section 3.4.

**Figure 1:**
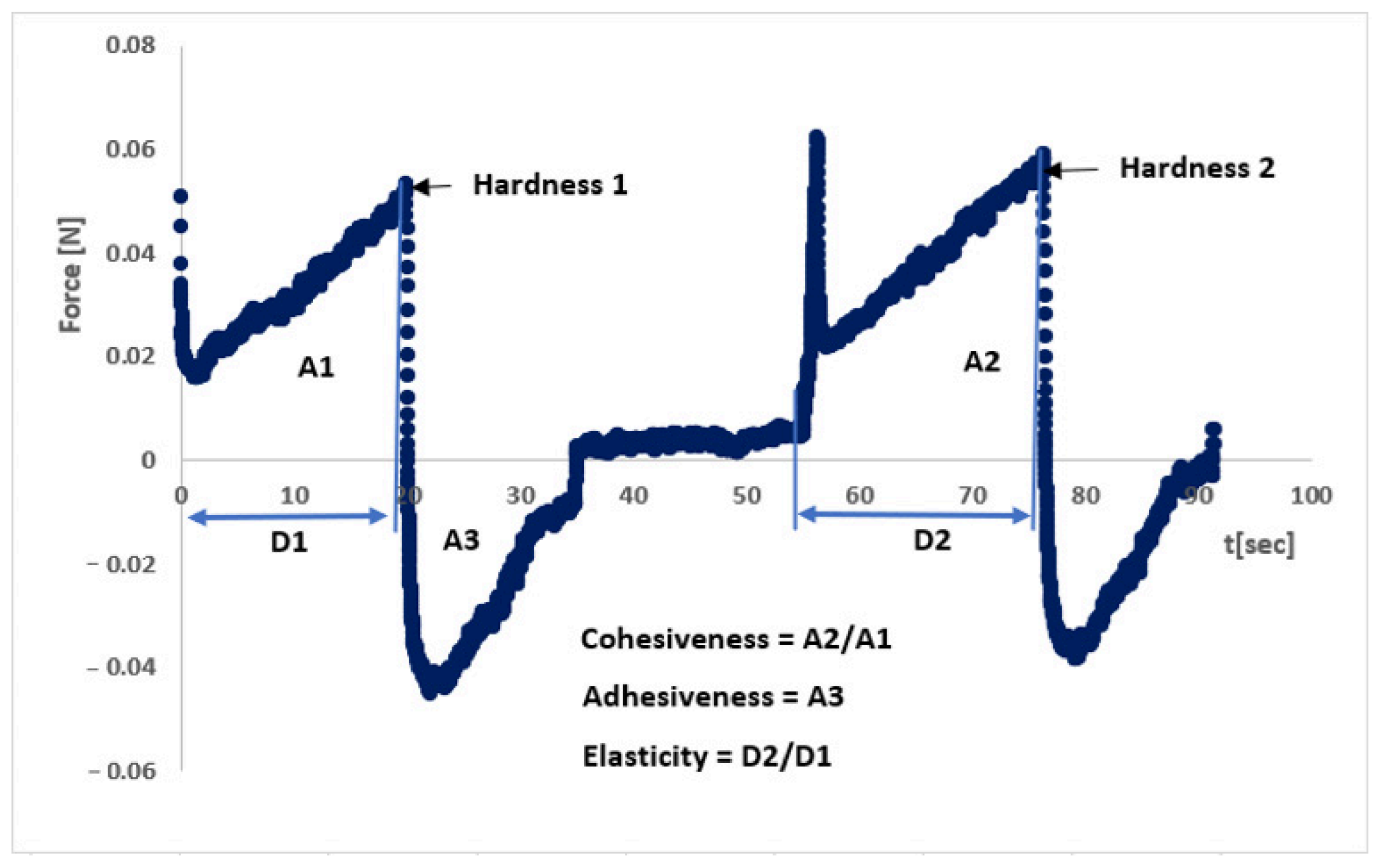
An example texture profile (TPA) of CU10/SA (1:2) hybrid hydrogel: soluble (Actrapid^®^) (Savitzky–Golay filtered profile).

**Figure 2:**
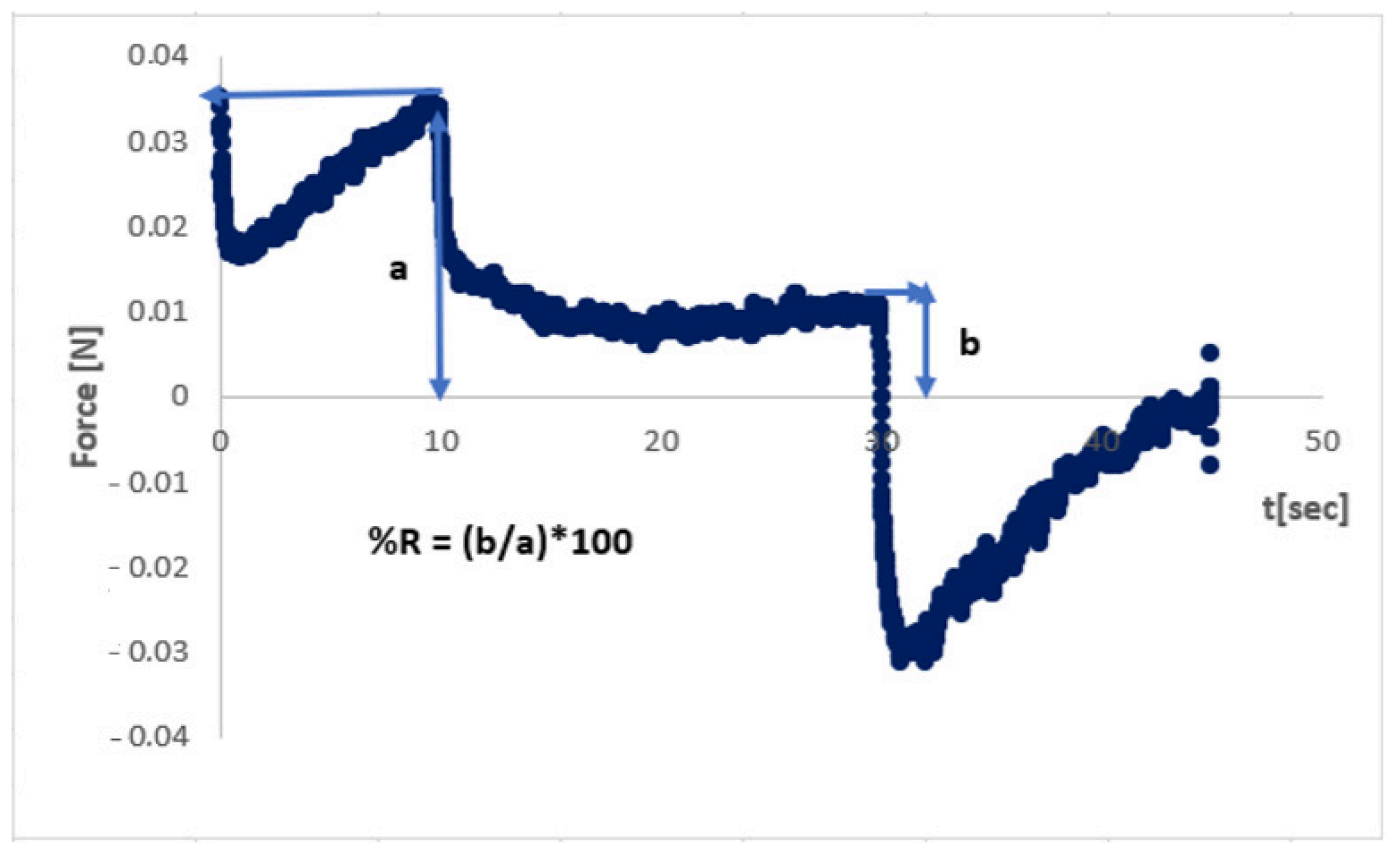
An example penetration test (CRT) of CU10/SA (1:2) hybrid hydrogel: soluble (Actrapid^®^) (Savitzky–Golay filtered profile).

The results of the texture analysis (Table 1, Figure 1 and Figure 2) for the CU10/SA—INS solution (Actrapid^®^) and the CU10/SA—INS suspension (Insulatard^®^) suggest that incorporating an insulin solution or suspension into the hydrogel matrix does not significantly affect the mechanical properties of the preparation (*p* = NS). The form of insulin did not significantly affect cohesion (internal cohesion) or adhesion. However, insulin form affected relaxation [%], which was higher in the CU10/SA–INS solution (Actrapid^®^) system. Relaxation in physically cross-linked hydrogels results from viscoelastic effects corresponding to polymer-chain rearrangement [34]. This parameter regulates the rate of API diffusion from the matrix, in accordance with the principle that greater stress relaxation leads to faster polymer-network unraveling under a constant applied force. The lower relaxation of the suspension gel (83.0% vs. 87.3%, *p* < 0.01) therefore points to a network that unravels more slowly under load, which is consistent with its higher viscosity (Table 1), its lower flow behavior index (Section 3.4), and its lower cumulative release (Section 3.2). The lower relaxation of the Insulatard^®^ insulin-containing hydrogel may potentially be due to the presence of zinc protamine crystals, which may limit the reorganisation of the polymer network under load.

In our previous texture analysis of the CU10–INS solution (Actrapid^®^) hydrogel, the texture parameters were as follows: relaxation 54.2 ± 1.02%, hardness1 0.061 ± 0.02 N/m^2^, hardness2 0.069 ± 0.04 N/m^2^, cohesiveness 1.087 ± 0.02, adhesiveness 0.3 ± 0.01 mJ and elasticity 0.959 ± 0.06 [20]. Adding sodium alginate to the hydrogel formulation increased relaxation by 61%, cohesiveness by 24%, and elasticity by 9%. Adhesiveness remained at the same level. Hardness 1 and hardness 2 decreased by 11% and 19%, respectively. This suggests that the CU10/SA–INS system is more elastic and less rigid. These results are consistent with Khoobbakht et al. [35], confirming that combining synthetic and natural polymers improves the hydrogel’s mechanical properties. Adding locust bean gum (LBG) and xanthan gum (XG) to agar increased the springiness of the agar hydrogel and reduced its hardness. Consequently, the developed gel formulation was less rigid and more elastic than the agar hydrogel.

### 3.2. In Vitro Release Profiles and Kinetics

**Figure 3:**
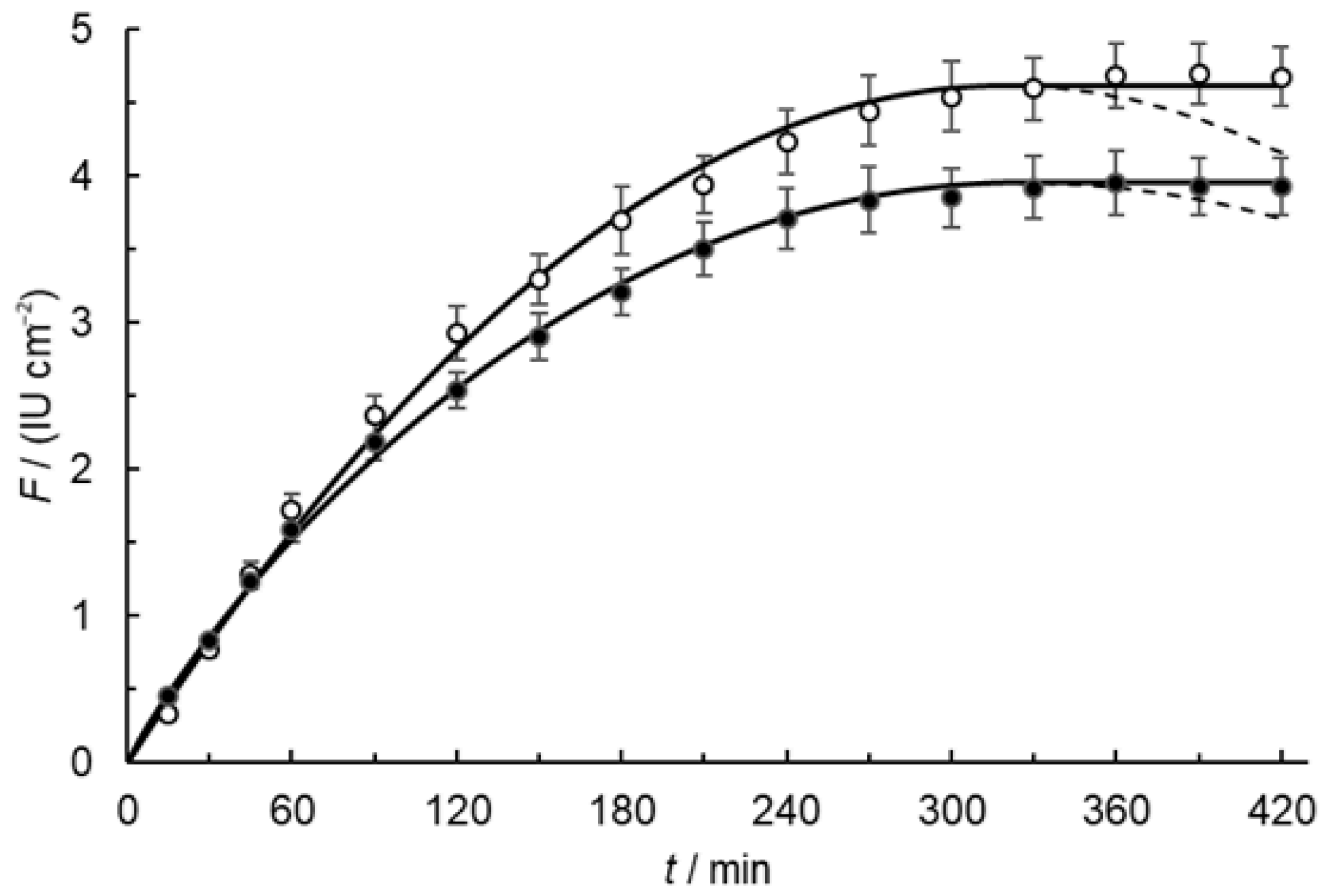
Cumulative insulin release per unit area of a Strat-M^®^ membrane from the CU10/SA (1:2) hybrid hydrogel: comparison between the solution (Actrapid^®^) and suspension (Insulatard^®^) formulations. Points represent experimental data for Actrapid^®^ (empty circles) and Insulatard^®^ (filled circles). Solid lines show the fit of the Peppas–Sahlin model (Equation (6)) for *t* < 330 min and constant values for t > 330 min. Dashed lines represent the model extrapolation beyond its range of applicability. Error bars indicate standard deviations.

Both formulations showed sustained cumulative release increasing over the 330 min period, with no burst effect (Figure 3). After that, the process ended, and the cumulative release value stayed constant for the remainder of the measurement run. Throughout the experiment, the soluble Actrapid^®^ released more insulin than the Insulatard^®^ formulation (4.67 IU/cm^2^ vs. 3.93 IU/cm^2^), i.e., the cumulative release from the suspension was 15.9% lower. During the first 30 min, the soluble formulation showed a slightly slower release; however, both profiles remained virtually indistinguishable within the margin of measurement uncertainty. Afterward, the profiles diverged progressively, with the soluble formulation releasing faster.

Several release kinetics equations were tested, including zero-order, first-order, Higuchi, Hixson–Crowell, Weibull, Korsmeyer–Peppas, and Peppas–Sahlin models [17,18,19,20]. The shape of the kinetics curves obtained in the present study (Figure 3) suggested that the Peppas–Sahlin model could correctly approximate the empirical behavior. The model is given by the following equation:

*F* = *k*_1_*t^m^* + *k*_2_*t*^2*m*^, (6)

where *F* is the cumulative insulin release per unit membrane area, *t* is time, and *k*_1_, *k*_2_, and *m* are the model parameters. In fact, this model is the only one from the seven studied previously [17,18,19,20] that accounts for an approximately linear increase in *F* in the initial stage and provides a smooth transition at *t* ≈ 330 min to the flat section of the curve. At *t* > 330 min, no further release occurs, and the model is not valid. Table 2 reports the fitted model parameters, and Figure 3 plots the corresponding curves. The model approximated both kinetic curves well, as shown by the high $R_{\mathrm{adj}}^{2}$ values. A slightly better fit was obtained for the suspension formulation.

**Figure 4:**
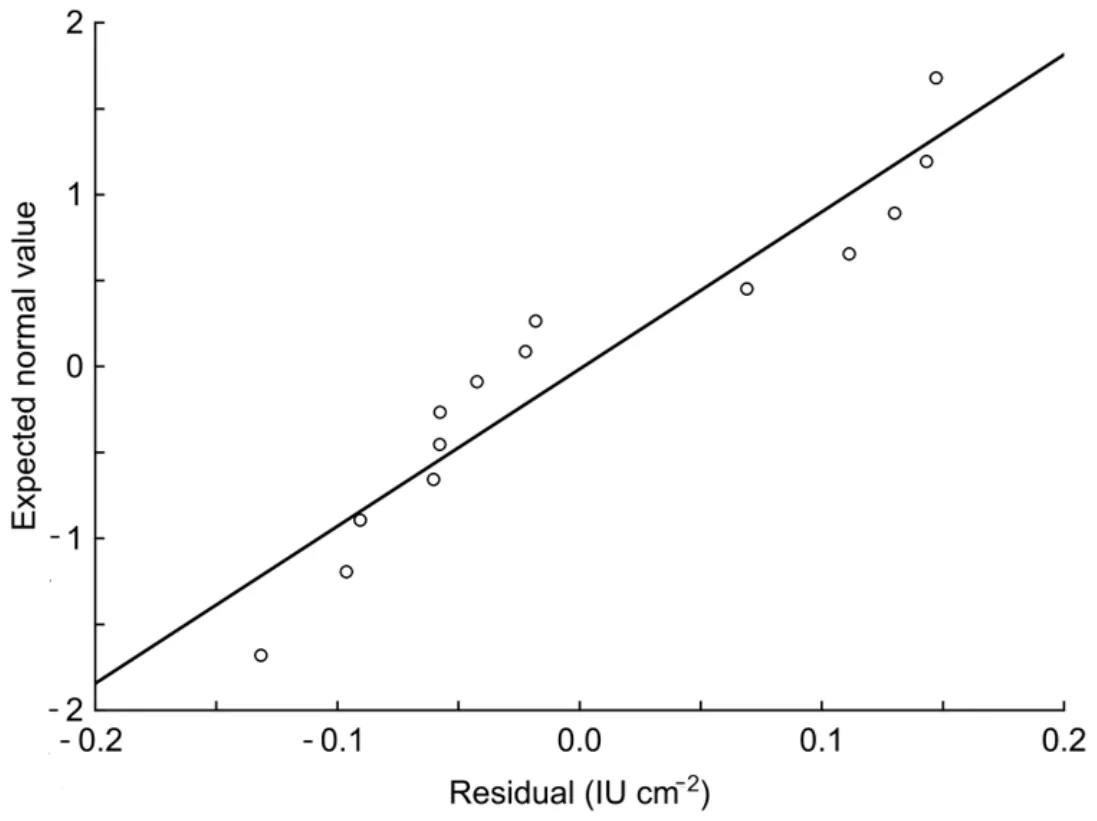
Normality plot of residuals for the CU10/SA (1:2) hybrid hydrogel: soluble (Actrapid^®^), obtained for the Peppas–Sahlin model.

**Figure 5:**
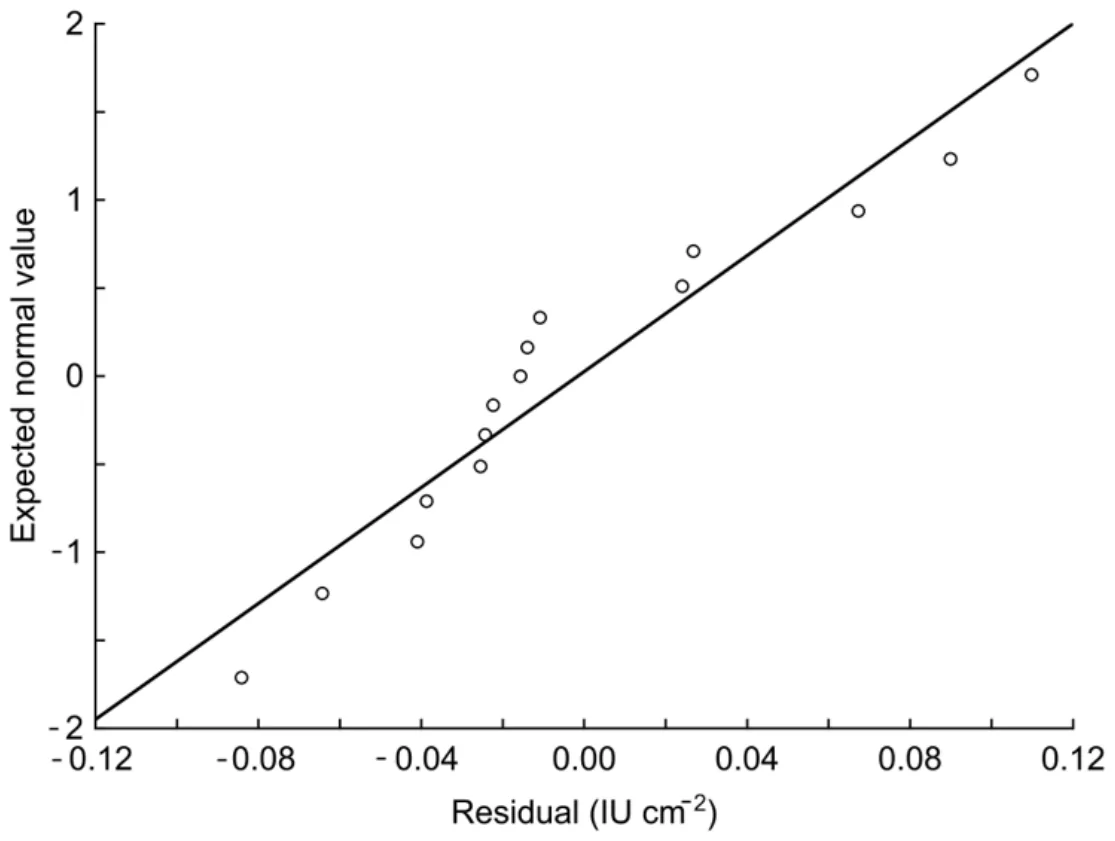
Normality plot of residuals for the CU10/SA (1:2) hybrid hydrogel: suspension (Insulatard^®^), obtained for the Peppas–Sahlin model.

The relationship *k*_1_ > *k*_2_ and the negative value of *k*_2_ suggest that Fickian diffusion dominates the observed process, with only a minor contribution from the polymer relaxation component [36]. The Peppas–Sahlins model provided a good fit to the data across the entire time range analyzed, as confirmed by the residual analysis for both samples studied (Figure 4 and Figure 5). A similar predominance of diffusion in insulin release from a hybrid hydrogel based on hydroxypropyl methylcellulose and sodium hyaluronate was observed previously [18]. Deang et al. [37] suggested that diffusion determines the kinetics of vitamin B12 release from an agar/κ-carrageenan hydrogel, albeit they applied the Korsmeyer–Peppas model rather than the Peppas–Sahlin one.

**Figure 6:**
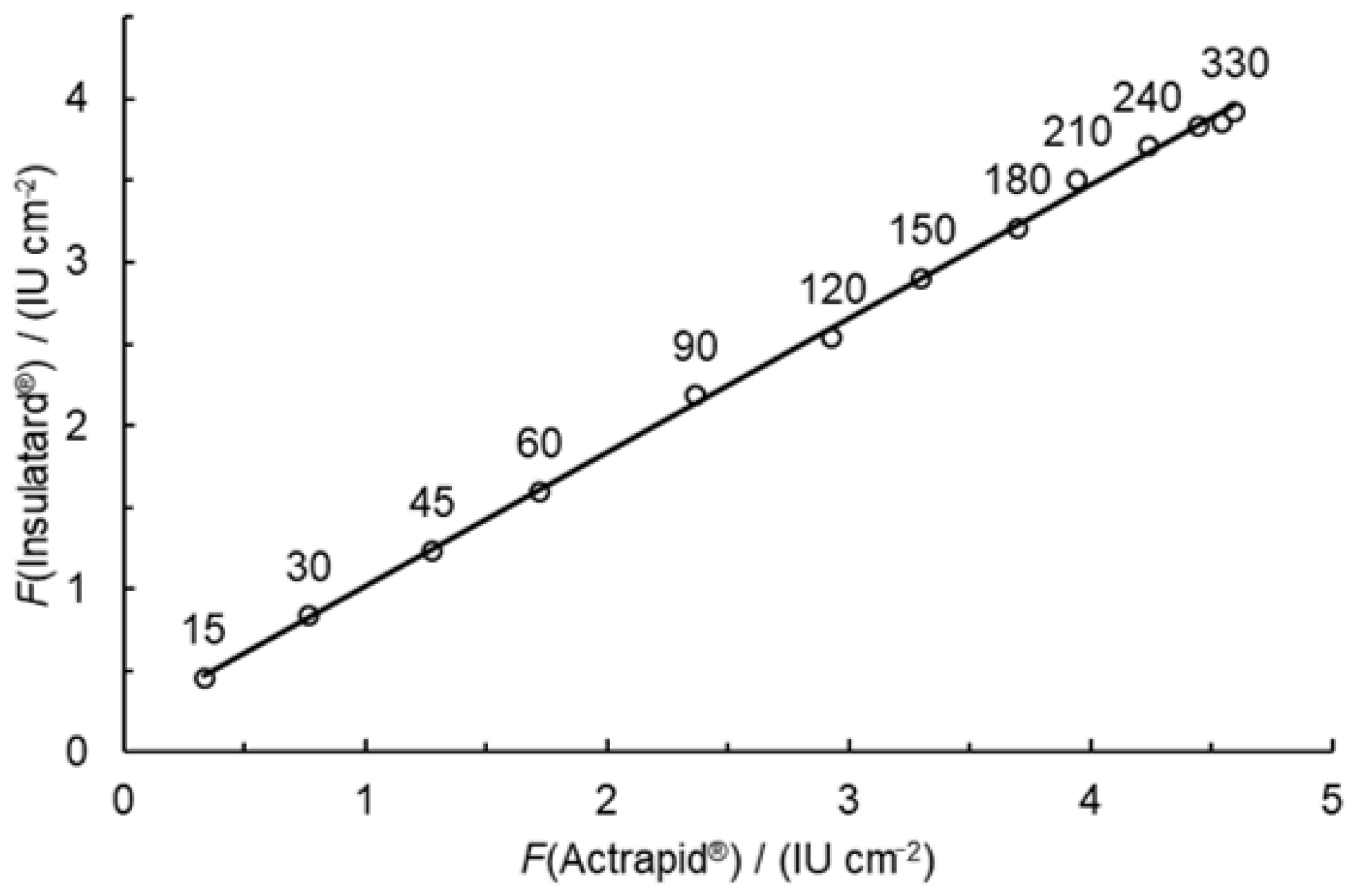
Correlation between the values of cumulative insulin release from the CU10/SA (1:2) hybrid hydrogel: soluble (Actrapid^®^) versus suspension (Insulatard^®^) formulations. The point labels show the time (in minutes) of the concentration measurement.

The two release profiles are similar. The difference factor *f*_1_ = 13.39 fell within the 0–15 range accepted by the FDA and EMA, and the similarity factor *f*_2_ = 97.54 significantly exceeded the threshold value of 50. A simple regression line between the two datasets plotted in Figure 6 illustrates the similarity. The only significant difference is in the extent of release.

The same conclusion follows from the areas under the release curves from *t* = 0 to 330 min, which were 998 and 881 IU·min·cm^−2^ for the solution and the suspension, respectively, indicating a 12% difference.

### 3.3. Molecular Docking of Insulin–Polymer Interactions

This section examines whether the observed macroscopic behavior of the systems can be related to interactions at the molecular level. Molecular docking was conducted to address a specific inquiry arising from the release data. The release kinetics were consistent with a predominantly diffusion-controlled process rather than with erosion-controlled release. The docking therefore aimed to identify interactions that might slow insulin diffusion, to assess whether they are weak and reversible, as sustained release would require, and to determine whether the two polymers of the blend interact with insulin in a similar way. These results are interpreted together with the release data (Section 3.2) in Section 3.5.

**Figure 7:**
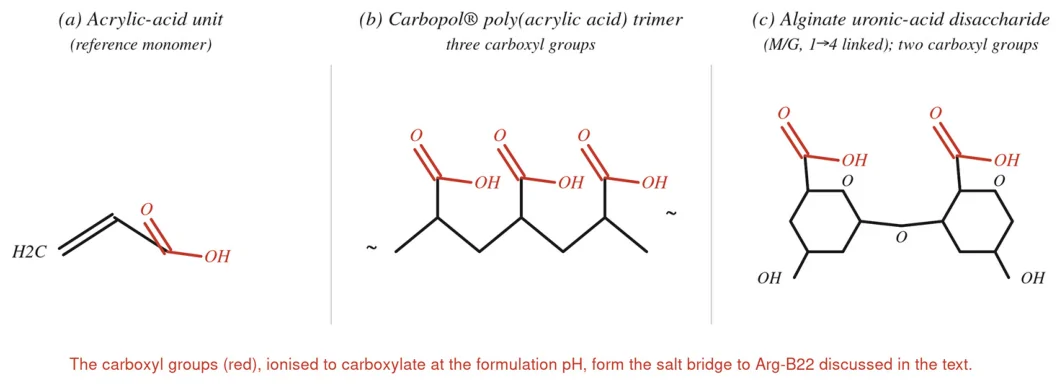
Structural formulas of the three polymer fragments used as docking ligands, generated in ChemDraw 26.0 (Revvity Signals Software, Waltham, MA, USA). Panel (**a**) shows the acrylic-acid reference unit, panel (**b**) the Carbopol poly(acrylic acid) trimer, and panel (**c**) the alginate uronic-acid disaccharide. The carboxylate groups that form the salt bridge to Arg-B22 are marked. The arrow in panel (**c**) denotes the 1→4 glycosidic linkage between the two uronic-acid residues.

Blind docking detected five surface cavities on the human insulin monomer for each ligand. The best (lowest-energy) docking scores were −4.9 kcal/mol for the alginate disaccharide, −4.5 kcal/mol for the Carbopol trimer, and −2.7 kcal/mol for the single acrylic-acid unit (Table 3, Figure 7). Notably, the top-ranked cavity for both the alginate disaccharide and the Carbopol trimer was the same B-chain pocket (cavity C2, center −18, 7, 0 Å), suggesting that the two polymers converge on a common region of the insulin surface. The weaker score of the acrylic-acid unit largely reflects its small size (five non-hydrogen atoms). The trimer, which carries three carboxylate groups, is a more representative model of the poly(acrylic acid) backbone of Carbopol and gives a value close to that of alginate. On a per-atom (ligand-efficiency) basis, the values were approximately −0.54 kcal/mol per non-H atom for acrylic acid, −0.30 for the Carbopol trimer, and −0.20 for alginate. The high value for acrylic acid should be treated with caution, because ligand efficiency tends to favor very small ligands [38].

**Figure 8:**
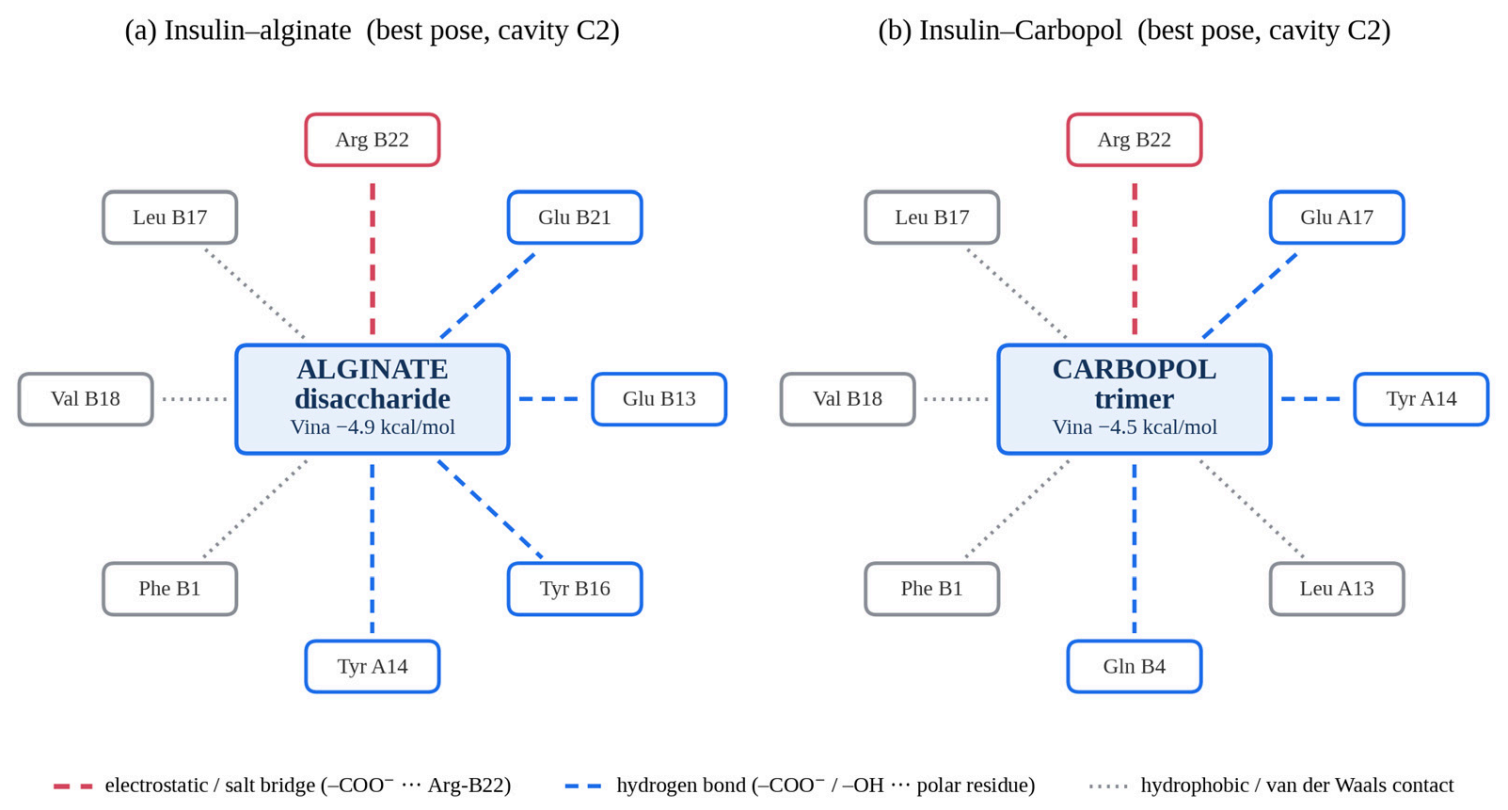
Predicted contact residues and interaction types for the best insulin–alginate (**a**) and insulin–Carbopol (**b**) poses. Red = electrostatic/salt-bridge; blue = hydrogen bond; grey = hydrophobic/van der Waals. In the legend, dots (···) denote a non-covalent contact between the polymer group and the insulin residue, and dotted grey lines denote hydrophobic or van der Waals contacts.

Analysis of the best poses suggested that both polymers engage insulin through the same type of interactions and, on human insulin, at the same site, namely through electrostatic (salt-bridge) contacts between their deprotonated carboxylate groups and the basic residue Arg-B22, reinforced by hydrogen bonds to neighboring polar and acidic residues (Glu-B21, Glu-B13, Tyr-B16 and His-B10 for alginate; Glu-A17 and Tyr-A14 for the Carbopol trimer) and supported by hydrophobic contacts with Phe-B1, Val-B18 and Leu-B17 common to both (Figure 8, Table 4). Both the alginate disaccharide and the Carbopol trimer docked into the same B-chain pocket (cavity C2), with the carboxylate–Arg-B22 salt bridge as the predicted dominant anchoring feature in each case. This convergence of the two carboxylated polymers of the blend on a common, positively charged insulin pocket offers a plausible molecular picture of how the matrix could retard insulin diffusion, and this was treated as a hypothesis to be tested rather than an established fact.

**Figure 9:**
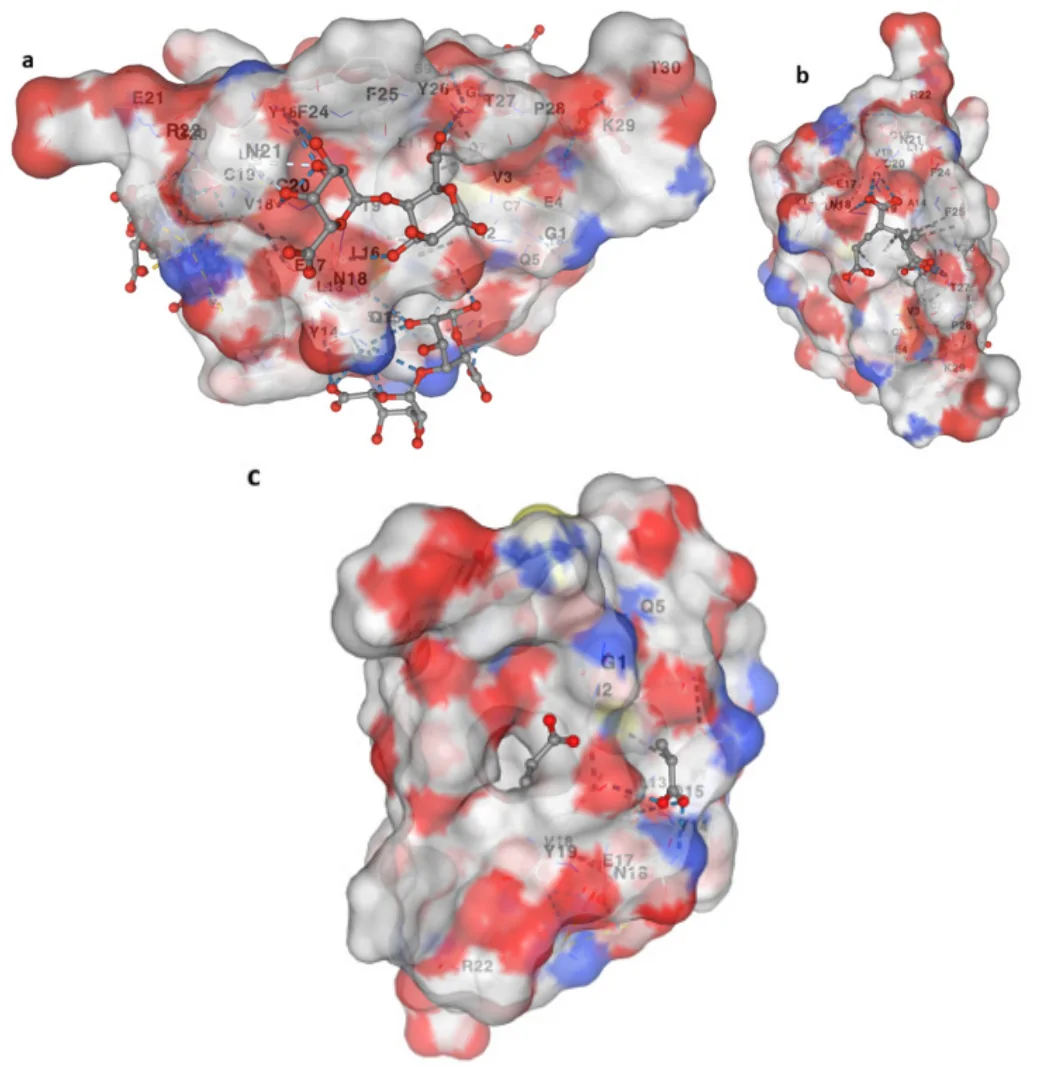
Three-dimensional docked poses on human insulin (PDB 1MSO; CB-Dock2/AutoDock Vina, best cavity) of (**a**) the alginate disaccharide (−4.9 kcal/mol), (**b**) the Carbopol trimer (−4.5 kcal/mol), and (**c**) the single acrylic-acid unit (−2.7 kcal/mol). The insulin surface is colored by atom type (red = oxygen, blue = nitrogen, white = carbon, yellow = sulfur). Ligands are shown as ball-and-stick models (grey = carbon, red = oxygen). Dashed lines show predicted interactions (blue = hydrogen bonds, yellow = salt bridges, grey = hydrophobic contacts), and residues are labeled with one-letter codes. In the predicted poses, all three ligands localize to the same B-chain surface region, with the carboxylate–Arg-B22 salt bridge as the main anchoring contact (the acrylic-acid unit serves as a size-limited reference).

Figure 9 shows the 3-D docked poses on human insulin, and Table 4 lists the complete set of human insulin residues in contact with each polymer at its best-scoring cavity. In the predicted poses, the polymer carboxylates insert into a shallow, positively charged B-chain surface pocket and are anchored primarily by Arg-B22, with surrounding acidic and polar residues (Glu-B21, Glu-B13, Glu-A17, Tyr-B16, and His-B10, depending on the fragment) forming the hydrogen-bond network and hydrophobic residues (Phe-B1, Val-B18, Leu-B17) lining the pocket.

#### Robustness to Insulin Assembly State

To test whether the identified interaction is an artifact of using an isolated monomer, both fragments were re-docked against the assembled insulin hexamer. Both again localized to the same B-chain surface region. The alginate disaccharide gave a best score of −7.4 kcal/mol at cavity C2, engaging Tyr-B16, Gly-B20, Glu-B21 and Arg-B22 (with His-B10 and adjacent-subunit residues lining the inter-dimer cleft), and the Carbopol trimer gave −3.9 kcal/mol contacting His-B10, Glu-B13, Tyr-B16, Glu-B21 and neighboring residues of the same patch. The scores are not directly comparable between the two receptors. The alginate fragment scored more favorably on the hexamer (−7.4 vs. −4.9 kcal/mol), consistent with the deeper inter-subunit clefts of the assembly, whereas the smaller Carbopol trimer scored less favorably (−3.9 vs. −4.5 kcal/mol).

**Figure 10:**
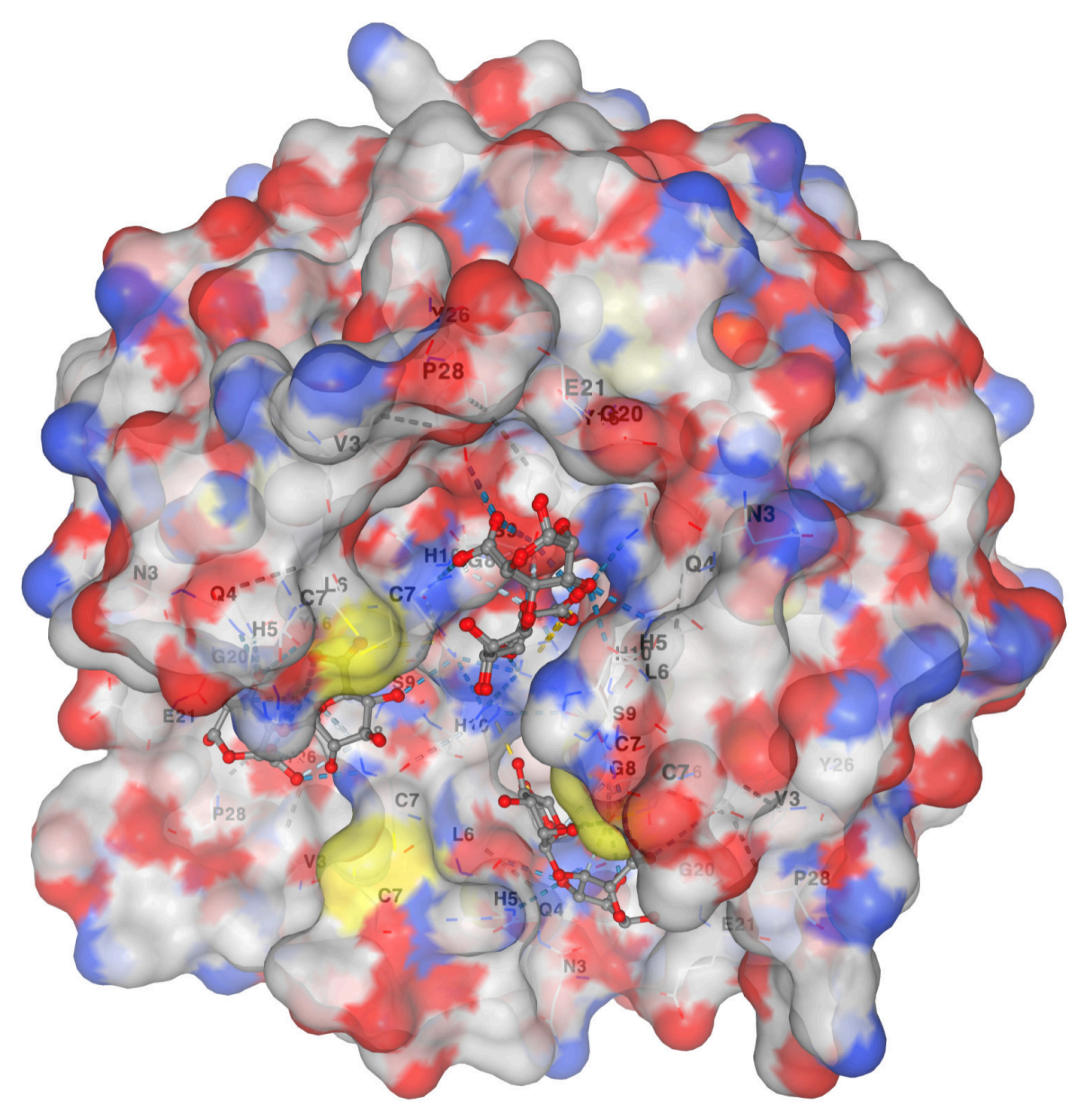
Alginate disaccharide docked onto the solvent-accessible surface of the insulin hexamer (PDB 1MSO; CB-Dock2/AutoDock Vina, best cavity, −7.4 kcal/mol). The insulin surface is colored by atom type (red = oxygen, blue = nitrogen, white = carbon, yellow = sulfur), and the ligand is shown as a ball-and-stick model. Dashed lines show predicted interactions (blue = hydrogen bonds, yellow = salt bridges, grey = hydrophobic contacts), and residues are labeled with one-letter codes. Poses in the symmetry-related cavities C4 and C5 (Table 5), which share the same contact residues, are also shown; labels indicate contacting insulin residues. The fragment localizes to the same B-chain patch (His-B10, Tyr-B16, Gly-B20, Glu-B21, and Arg-B22) identified on the monomer, indicating that the site remains accessible on the assembled hexamer.

Because raw docking scores increase with ligand size, the fragments were compared on the basis of ligand efficiency, defined as the docking score divided by the number of non-hydrogen atoms, although this normalization has recognized limitations [38]. For the monomer, the ligand efficiencies of alginate and Carbopol were approximately −0.20 and −0.30 kcal/mol per non-hydrogen atom, respectively, whereas for the hexamer the corresponding values were approximately −0.30 and −0.26 kcal/mol. The higher efficiency of alginate on the hexamer is consistent with the deeper inter-subunit clefts of the assembled receptor, which accommodate more of the disaccharide and increase its buried surface area. This difference is therefore attributed to the assembly’s surface geometry rather than to a stronger salt bridge, since both polymers localize to the same B-chain acidic patch and the alginate fragment engages Arg-B22 on both receptors. The binding-site location, rather than the score magnitude, is conserved between the two receptors, and the more favorable raw score for the hexamer is not interpreted as evidence of tighter binding. The Arg-B22/acidic-patch site therefore appears to be accessible in both the monomeric and the hexameric form of insulin (Figure 10).

Two limitations of the hexamer calculation should be noted. First, the zinc ions were removed together with the crystallographic water molecules, so the electrostatic environment of the central channel is not accurately represented. The favorable score for cavity C1, located within this channel and not involving Arg-B22, is therefore likely to be an artifact of zinc removal and is not interpreted. Second, cavity C1 has a CurPocket volume of 18,991 Å^3^ (Table 5) and thus corresponds to an extensive surface region rather than a discrete binding pocket. The interpretation consequently focuses on cavities C2–C5, all of which engage Arg-B22 in the alginate calculation (Table 5).

### 3.4. Rheological Properties of the Hydrogels

**Figure 11:**
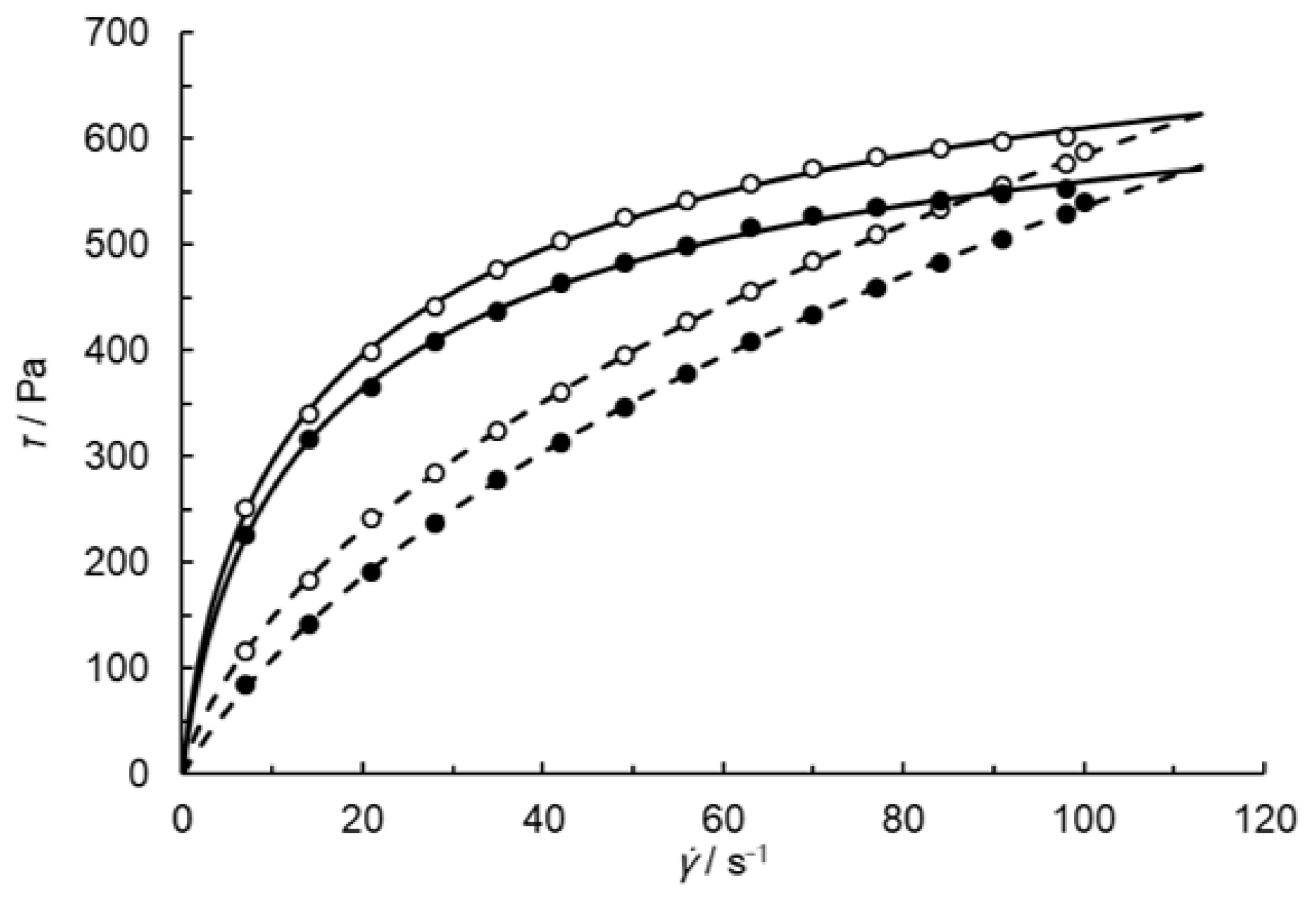
Shear rate vs. shear stress curves for two CU10/SA hydrogels containing Actrapid^®^ solution and Insulatard^®^ suspension at 25 °C. Points represent experimental results (empty circles: Actrapid^®^, filled circles: Insulatard^®^); lines represent the CY model (Equation (7)) for increasing (solid lines) and decreasing (dashed lines) shear rates. The SRB model (Equation (8)) yields virtually identical graphs.

The two CU10/SA insulin hydrogels are pseudoplastic fluids with pronounced shear-stress hysteresis (Figure 11). Two rheological models fairly well described the recorded shear stress vs. shear rate curves: the Carreau–Yasuda (CY) model [39,40] and the shear rate-based model (SRB) of Santesarti et al. [39]. The CY and SRB model equations were applied in the following forms:

(7) $$\tau =\dot{\gamma }\left\{\eta_{\infty }+\left(\eta_{0}-\eta_{\infty }\right){\left[1+{\left(\lambda \dot{\gamma }\right)}^{a}\right]}^{\frac{n-1}{a}}\right\}$$

and

(8) $$\tau =\dot{\gamma }\eta_{0}{\left[\frac{1+{\left(\lambda_{\infty }\dot{\gamma }\right)}^{a}}{1+{\left(\lambda_{0}\dot{\gamma }\right)}^{a}}\right]}^{\frac{1-n}{a}}$$

where *τ* is shear stress (Pa), $\dot{\gamma }$ is the shear rate (s), and *a* and *n* are dimensionless parameters; *a* adjusts the transition between the quasi-Newtonian and the dominant shear-thinning ranges (*a* > 1), and *n* regulates the shear-thinning rate, 0 < *n* < 1. *λ* is a relaxation time constant, i.e., “consistency”; its reciprocal value in the CY model characterizes the onset of significant shear-thinning effect. *η*_0_ and *η*_∞_ denote apparent viscosity at zero and infinite shear rate, respectively. Thus, the CY model applies two viscosity parameters (*η*_0_ and *η*_∞_) and one consistency parameter *λ*. The SRB model contains two-time constants, *λ*_0_ and *λ*_∞_, and only one viscosity parameter *η*_0_. Table 6 reports the two model parameters. Unfortunately, fitting the two models yielded degenerate solutions. Degenerate solutions indicate limited identifiability of the model parameters within the range of shear rates under investigation, where different parameter values can lead to similar fits of the experimental data. This is a known disadvantage of the CY model [39]. Santesarti et al. [39] suggested an elegant way of getting reasonable starting parameters for fitting the SRB model. However, the range of shear rates in our measurements was too narrow to follow that rule. Values of $R_{\mathrm{adj}}^{2}$, AIC, and MSC reported in Table 6 showed that the two models approximated the shear-stress dependence on shear rate equally well.

**Figure 12:**
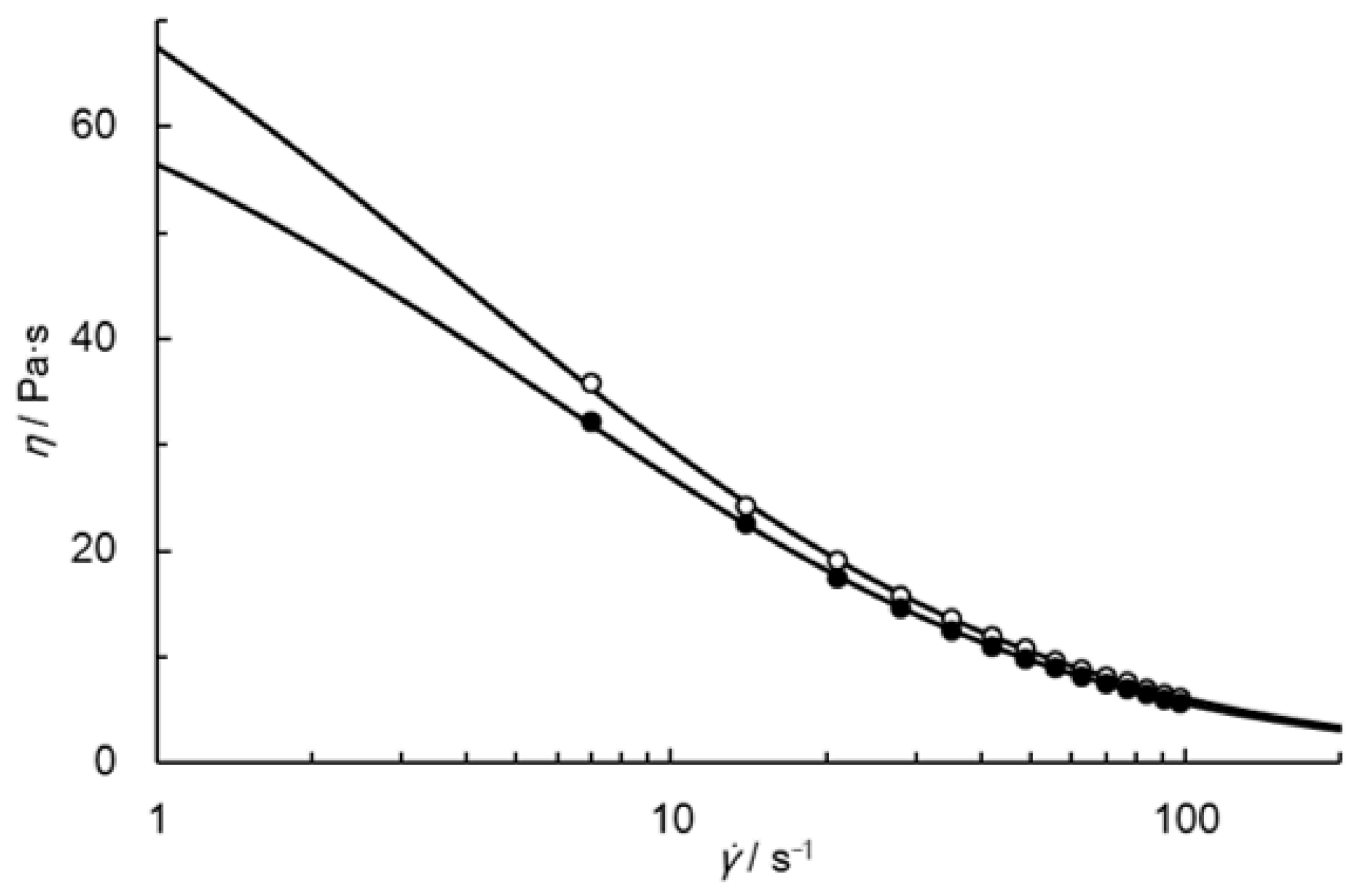
Shear rate vs. viscosity curves for two CU10/SA hydrogels containing Actrapid^®^ solution and Insulatard^®^ suspension at 25 °C. Points represent experimental results (empty circles: Actrapid^®^, filled circles: Insulatard^®^); lines represent the CY model (Equation (7)) for increasing shear rates. The SRB model (Equation (8)) yields similar graphs.

Since viscosity is defined as:

(9) $$\eta \left(\dot{\gamma }\right)=\tau /\dot{\gamma },$$

it can easily be calculated from the model Equations (2) and (3) for any shear rate. The viscosity curves are plotted in Figure 12. Within the range of shear rates investigated, no clear quasi-Newtonian region was identified, where *η* ≈ const. Indeed, the reciprocal relaxation time constants *λ*^−1^ assessed from the CY↑ model curves (Table 6) are approximately equal to 0.09 s^−1^. This value is much smaller than the lowest shear rate of 7 s^−1^ attained in the measurements. Although the hydrogels visually resembled Bingham plastics, a value of yield stress could not be assessed for the same reason. A “yield stress parameter” can be easily introduced into model Equations (7) and (8) as the intercept coefficient. The modified equations could interpolate the experimental dependencies at least equally well. However, it was not possible to extend the analysis to lower shear rates due to the lack of stable and reproducible measurement results in this range $(\dot{\gamma }<7s^{-1}$). Consequently, the analysis was carried out in the shear rate range of 7–100 s^−1^, corresponding to the range in which stable measurement results were obtained. The parameters of the rheological models, including η_0_ and λ, were interpreted in the context of the available experimental data and the model used.

The shear-thinning of the studied hydrogels results from particle reorganization under shear stress. As the shear rate increases, asymmetrical particles align with the flow axis, reducing viscosity. This allows the hydrogels to spread easily on the skin during application while maintaining structural integrity within the unit packaging [41,42]. The low viscosity of both formulations, *η* < 10 Pa·s at a moderate shear rate of 100 s^−1^ (Table 1), is highly desirable for practical use [43]. Moreover, thixotropic hydrogels easily spread at the site of application and thicken at rest.

The area within the hysteresis loop quantitatively measures the thixotropic effect. This area is proportional to the ratio of the energy dissipated during material recovery after deformation to the total energy required for deformation [44,45]. Based on the CY model (Equation (7)), the calculated areas are 11.0 kPa/s and 11.7 kPa/s for the hydrogels containing Insulatard^®^ suspension and Actrapid^®^ solution, respectively. The area of the hysteresis loop varied by 5.7% between the samples analysed. This difference indicates that the thixotropic behaviour of the samples under investigation was comparable under the measurement conditions used. Consequently, both hydrogels dissipate approximately the same amount of structural energy, demonstrating rheological consistency.

### 3.5. A Molecular Basis for Diffusion-Controlled Release

The kinetic analysis (Section 3.2) indicates that insulin release from the CU10:SA matrix is predominantly diffusion-controlled. Insulin therefore appears to diffuse through a hydrated polymer network that transiently retards it, rather than being released by matrix erosion. The docking study suggests a possible interaction motif that could account for that retardation. At the formulation pH (~7.0–7.2), the carboxyl groups of both alginate and Carbopol are largely ionized (–COO^−^), and the docking suggests that they can form reversible electrostatic and hydrogen-bonding contacts with insulin, in particular a salt bridge to the basic residue Arg-B22, on which both polymer models converge. Interactions of this magnitude (best scores of −4.5 to −4.9 kcal/mol for the two polymer models) are consistent with weak, reversible physisorption rather than tight binding, which is the regime expected for a system whose release is diffusion-limited and sustained rather than irreversibly retentive.

Docking was performed first on the insulin monomer, on the reasoning that a diffusing polymer segment encounters the solvent-accessible surface of insulin rather than its interior, and then repeated on the zinc-stabilized hexamer that predominates in these zinc-containing formulations (see the Robustness to Insulin Assembly State Section). The B-chain pocket anchoring the carboxylate–Arg-B22 salt bridge is surface-exposed in both receptors and was engaged by the alginate fragment in both, so the proposed interaction does not appear to depend on the assumed association state. Experimental binding measurements, or molecular dynamics with explicit solvent and physiological ionic-strength (Debye) screening, were beyond the scope of the present work and would be needed to place the interaction on a firmer, more quantitative footing. These were identified as the appropriate next steps. The effect of physiological ionic strength on these electrostatic contacts is considered in the Influence of Physiological Ionic Strength section below.

#### Influence of Physiological Ionic Strength

The docking calculations were performed without explicit salt, whereas the hydrogels are intended for application to wounds where the surrounding fluid has high ionic strength. Wound exudate is derived from serum and has a comparable electrolyte composition, and the standard fluid used to simulate it contains approximately 142 mmol/L sodium [46]. At this ionic strength, the Debye screening length is only about 0.8 nm [47], which is comparable to the length of the carboxylate–Arg-B22 contact itself. The electrostatic attraction at this site is therefore expected to be considerably weaker at the wound site than the salt-free docking calculation suggests. However, the hydrogels in this study were prepared in PBS, and release was measured in PBS (pH 7.4), whose ionic strength (approximately 0.16 mol/L) is close to that of wound exudate. The measured release profiles therefore already reflect screened electrostatic interactions, and ionic strength alone is not expected to accelerate insulin release at the wound site substantially beyond the rate observed in vitro. These considerations further support interpreting the insulin–polymer interaction as weak and reversible rather than a strong anchor. Other exudate components absent from PBS, such as proteins, proteolytic enzymes, and divalent cations, may still influence release in vivo and warrant investigation in simulated wound fluid.

### 3.6. Alginate Versus Carbopol Interactions

On an equivalent-fragment basis, alginate (−4.9 kcal/mol) and Carbopol (−4.5 kcal/mol as the realistic trimer) are comparable in strength, within docking-score uncertainty, and both occupy the same Arg-B22 pocket (cavity C2). This is consistent with the molecular structure, i.e., the alginate disaccharide presents two carboxylates plus multiple hydroxyl groups capable of hydrogen bonding, whereas the poly (acrylic acid) fragment presents a dense array of carboxylates. Both are predicted to be anchored by the same Arg-B22 salt bridge. Because alginate is the major component of the 1:2 blend, it is likely to provide most of the carboxylate contacts available to insulin, with Carbopol contributing additional carboxylate density at the same site. The much weaker score of the single acrylic-acid unit (−2.7 kcal/mol) largely reflects its small size and illustrates why an oligomeric model is required for a fair polymer-to-polymer comparison. Molecular docking uses the same insulin structure for both formulations and therefore yields one interaction map, not two different binding energies. Docking describes a possible insulin–polymer interaction that could influence diffusion in general, but it does not predict, and should not be presented as predicting, a different affinity for Actrapid^®^ than for Insulatard^®^.

### 3.7. Why Is the Release from Suspension Slower?

Because the same insulin structure was used in the docking for both formulations, the docking cannot explain the lower release of insulin from the Insulatard^®^ suspension than from the Actrapid^®^ solution. This difference is more plausibly related to the pharmaceutical form of insulin and to the different composition of the two products. Insulatard^®^ is an isophane (NPH) preparation in which insulin is co-crystallized with protamine and zinc into microcrystalline particles [48]. Protamine is an arginine-rich, polycationic peptide that binds insulin electrostatically through its guanidinium groups; the resulting complex is larger, less soluble, and considerably less mobile than soluble insulin. Within the hydrogel, the microcrystalline insulin is likely to be sterically confined and physically entrapped, which would slow its dissolution and diffusion and lower its cumulative release. The similar shapes of the release profiles (Figure 3 and Figure 4; f1 = 13.39 and f2 = 97.54) suggest that a similar diffusion-controlled process operates in both gels, with the difference lying mainly in the extent of release. This interpretation is consistent with conclusions that differences in API release across formulations are driven by matrix hydration, viscosity, and osmotic factors rather than specific binding [18].

Beyond the difference in particle mobility, at least two additional, non-exclusive mechanisms could contribute to the slower release of the NPH suspension. First, protamine is a strongly cationic, arginine-rich peptide, and sodium alginate is a polyanion; the two can undergo polyelectrolyte complexation, so protamine associated with the NPH crystals may form additional ionic cross-links with the alginate matrix, locally densifying the network and further retarding insulin diffusion. In future studies, it would be appropriate to use spectroscopic methods, such as FTIR, to verify potential interactions between the components of the system under investigation. Second, the zinc present in both insulin formulations is a divalent cation that can ionically cross-link alginate, analogous to Ca^2+^, so differential zinc availability could modulate local matrix stiffness and mesh size. None of these mechanisms is accounted for in the model of insulin docking to the oligomer, which addresses only the direct insulin–polymer interaction; however, both are consistent with the diffusion-controlled release observed in these experiments. Seemingly, the stronger attraction forces in the Insulatard^®^ gel than in the Actrapid^®^ one should not lead to its lower viscosity and higher susceptibility to shear-thinning. However, microheterogeneities in the suspension gel most probably disrupt its cohesion. This phenomenon is less pronounced in a more homogeneous solution gel.

### 3.8. Context and Limitations

The predicted docking scores are modest. For comparison, docking of the much larger natural cyclodextrins to insulin gave scores of approximately −8 to −10 kcal/mol [49], so the weaker values obtained here for small polymer fragments are in line with transient rather than tight association. This is consistent with the interpretation of weak, reversible physisorption appropriate to a sustained-release system. The interaction modes suggested here, namely carboxylate-mediated electrostatic attraction and hydrogen bonding to insulin, mirror the interaction modes proposed, on experimental grounds, for other insulin–polyelectrolyte systems. Studies of insulin with polyacrylic acid-based carriers and interpolymer complexes (e.g., HPMC/poly (acrylic acid) buccal films) attribute drug retention to hydrogen bonding and electrostatic complexation between polymer carboxyls and insulin, and spectroscopic studies of insulin with natural biopolymers likewise identify hydrogen bonding and charge interactions as the dominant forces [50,51]. For the suspension, protamine, an arginine-rich polycation, is expected to associate with insulin mainly through electrostatic contacts with its acidic residues, which is consistent with the reduced mobility of NPH insulin discussed in Section 3.7. This docking simulation therefore suggests a molecular-level interpretation consistent with, and complementary to, the experimental results reported in the literature.

Several limitations should be acknowledged. (i) The polymers were modeled using short oligomer fragments instead of complete chains or the cross-linked network; thus, absolute scores should be interpreted as indicative of interaction tendencies rather than precise affinities. (ii) Blind docking with AutoDock Vina treats the receptor as essentially rigid and yields semi-quantitative scores. Docking short, highly charged polyanionic fragments without explicit solvent, counterions, or physiological ionic strength is also particularly prone to electrostatic artifacts, so the Arg-B22 contact should be regarded as a potential interaction motif rather than as established molecular evidence. (iii) A hydrogel is a hydrated, entangled network with transport behavior more complex than that of an isolated insulin–fragment pair. (iv) Only a single insulin conformation (PDB 1MSO) was utilized. (v) Docking was performed on both the insulin monomer and the assembled zinc hexamer, and the carboxylate-binding B-chain patch (His-B10/Tyr-B16/Glu-B21/Arg-B22) was engaged in both, so the proposed interaction does not appear to depend on the insulin association state. The Vina scores remain semi-quantitative and do not account for ionic-strength screening or polymer flexibility beyond short oligomers. (vi) Both receptors were prepared zinc-free, so the electrostatics of the central channel of the hexamer are not faithfully represented, and the favorable score obtained there (cavity C1) should not be interpreted; neither calculation accounts for ionic (Debye) screening at physiological ionic strength. Consequently, the scores indicate interaction tendency, not quantitative affinity. (vii) The results obtained should be interpreted taking into account differences in the composition of the preparations analyzed. The influence of excipients in the insulin preparations, including protamine and Zn^2+^ ions, which may interact with hydrogel network components and alter its properties, cannot be ruled out. (viii)The hydrogels are hyperosmolar (≈936–953 mOsm/L), above physiological tonicity (~300 mOsm/L). A hyperosmolar carrier may aid wound cleansing, help control exudate, and slightly limit microbial growth [52], and is generally well tolerated on intact skin; on damaged or ulcerated skin, however, it may cause transient stinging and osmotic fluid shifts, so tolerance would need to be confirmed for chronic use.

## 4. Conclusions

Insulin release from the CU10:SA (1:2) hybrid hydrogel is a sustained, burst-free process controlled by Fickian diffusion. Molecular docking suggests a possible molecular basis for this behavior, pointing to two reversible interaction motifs. The first is a salt bridge between polymer carboxylates and Arg-B22, a site that remains accessible in both the insulin monomer and the assembled hexamer. The second is hydrogen bonding to neighboring polar and acidic residues. The docking scores for alginate and the Carbopol trimer were similar, at −4.9 and −4.5 kcal/mol, respectively.

These weak, reversible interactions would likely cause diffusion-limited retardation rather than tight retention. The docking cannot account for the greater release from the soluble Actrapid^®^ formulation than from the Insulatard^®^ suspension, because the same insulin structure was modeled for both, and this difference is more plausibly related to the pharmaceutical form of insulin. The protamine–zinc microcrystalline complex of NPH insulin is larger and less mobile, so it is likely to be entrapped more effectively in the matrix. Rheological and textural data support this picture. Although the two gels show similar hardness, cohesiveness, adhesiveness, and elasticity, the Insulatard^®^ suspension gel is more viscous at both 30 s^−1^ and 100 s^−1^ and, at the same time, has a lower tendency to shear-thinning, which may be linked to its micro-heterogeneous structure.

## Figures and Tables

**Table 1:** Physicochemical characteristics of the insulin-loaded CU10/SA hydrogels (T = 25 °C ± 0.1 °C).

Parameter	CU10/SA—INS Solution (Actrapid^®^) Mean ± SD (*n* = 6)	CU10/SA—INS Suspension (Insulatard^®^) Mean ± SD (*n* = 6)	*p*
Insulin solution/NPH insulin suspension	Soluble monomer/hexamer	Protamine–zinc microcrystalline suspension (NPH)	-
pH	7.02 ± 0.01	7.16 ± 0.01	-
Osmotic pressure (mOsm/L)	936 ± 2	953 ± 2	-
Viscosity [Pa·s] at a shear rate of 30 s^−1^	7.0 ± 0.8 (25 °C)	7.9 ± 0.5 (25 °C)	<0.01
Viscosity [Pa·s] at a shear rate of 100 s^−1^	3.4 ± 0.6 (25 °C)	4.0 ± 0.3 (25 °C)	<0.01
Relaxation [%]	87.3 ± 0.9	83.0 ± 0.4	<0.01
Hardness 1 [N]	0.054 ± 0.002	0.055 ± 0.002	NS
Hardness 2 [N]	0.056 ± 0.009	0.057 ± 0.001	NS
Cohesiveness [−]	1.349 ± 0.123	1.338 ± 0.155	NS
Adhesiveness [mJ]	0.30 ± 0.04	0.3 ± 0.1	NS
Elasticity [−]	1.045 ± 0.062	1.045 ± 0.058	NS

NS—not statistically significant.

**Table 2:** Parameters of the Peppas–Sahlin model (Equation (6)) of the insulin release kinetics from the two CU10/SA hydrogels with their standard deviations, upper limit of the time domain of the model applicability *t*_max_, and the adjusted coefficient of determination $R_{\mathrm{adj}}^{2}$.

Parameter	Solution (Actrapid^®^)	Suspension (Insulatard^®^)
*k*_1_·10^2^/(IU cm^−2^ min^−*m*^)	2.89 ± 0.08	4.22 ± 0.20
*k*_2_·10^4^/(IU cm^−2^ min^−2*m*^)	–0.45 ± 0.03	–1.12 ± 0.11
*m*	1 ^a^	0.90 ± 0.01
*t*_max_/min	330	330
$R_{\mathrm{adj}}^{2}$	0.9958	0.9980

^a^ a constant value based on the result of *t*-testing.

**Table 3:** Docking scores for the insulin–polymer fragment complexes on human insulin (PDB 1MSO; CB-Dock2/AutoDock Vina; blind docking over the insulin monomer; deprotonated carboxylate ligands; five cavities per ligand).

Ligand Efficiency	Non-H Atoms	Cavity Range (kcal/mol)	Best Docking Score (kcal/mol)	Ligand
−0.20	25	−4.3 to −4.9	−4.9	Alginate disaccharide
−0.30	15	−3.7 to −4.5	−4.5	Carbopol trimer (3× COO^−^)
−0.54	5	−2.1 to −2.7	−2.7	Carbopol acrylic-acid unit

Note: The alginate disaccharide and the Carbopol trimer share the same best cavity (C2, center −18, 7, 0). Vina scores are empirical and semi-quantitative; values indicate interaction tendency, not measured binding free energies.

**Table 4:** Human insulin residues in contact with each polymer fragment at its best-scoring cavity (CB-Dock2 CurPocket output).

Contacting Human Insulin Residues (Best Pose)	Key Anchoring Residue	Best Cavity (Vina)	Ligand
A-chain: Leu13, Tyr14, Leu16, Glu17; B-chain: Phe1, Val2, Gln4, Leu6, His10, Glu13, Ala14, Leu15, Tyr16, Leu17, Val18, Cys19, Gly20, Glu21, Arg22	Arg-B22 (salt bridge)	C2 (−4.9)	Alginate disaccharide
A-chain: Leu13, Tyr14, Glu17; B-chain: Phe1, Val2, Gln4, Leu6, Ala14, Leu17, Val18, Arg22 (same pocket as alginate)	Arg-B22 (salt bridge)	C2 (−4.5)	Carbopol trimer
A-chain: Leu13, Tyr14, Glu17; B-chain: Phe1, Ala14, Leu17, Val18, Arg22 (size-limited reference fragment)	Arg-B22	C2 (−2.7; tied with C1)	Carbopol acrylic-acid unit

Note: In the predicted poses, all three ligands localize to the same B-chain surface pocket, and the carboxylate–Arg-B22 salt bridge is the predicted dominant anchoring interaction.

**Table 5:** Alginate–hexamer docking cavities (CB-Dock2/CurPocket). Cavities C2–C5 all engage Arg-B22, indicating convergence on the same site as on the monomer.

Key B-Chain Contacts	Volume (Å^3^)	Vina (kcal/mol)	Cavity
His10, Tyr16, Gly20, Glu21, **Arg22**	464	−7.4	C2
His10, Tyr16, Gly20, Glu21, **Arg22**	85	−7.4	C5
Glu13, Tyr16, Leu17, Gly20, Glu21, **Arg22**, Gly23	117	−7.2	C3
His10, Tyr16, Gly20, Glu21, **Arg22**	111	−7.2	C4
His10, Tyr16, Gly20, Glu21 (central Zn channel; no Arg22)	18,991	−7.0	C1

Note: Arg22 is shown in bold to highlight engagement of the Arg-B22 anchoring residue.

**Table 6:** Coefficients of the Carreau–Yasuda (CY, Equation (7)) and the shear rate-based (SRB, Equation (8)) model rheological curves for the two CU10/SA insulin hydrogels and statistical characteristics of the fit. Arrows after the model initialism denote the measurement runs: increasing (↑) and decreasing (↓) shear rate.

Parameter	Soluble Actrapid^®^				Suspension Insulatard^®^			
	CY↑	CY↓	SRB↑	SRB↓	CY↑	CY↓	SRB↑	SRB↓
*η*_0_/(Pa·s)	99.72	81.65	99.20	70.86	73.63	16.07	77.2	14.94
*η*_∞_/(Pa·s)	0	0	–	–	0	0	–	–
*λ*·10^2^/s	11.81	0.64	–	–	10.92	1.92	–	–
*λ*_0_·10^2^/s	–	–	11.82	3.40	–	–	10.40	1.55
*λ*_∞_·10^5^/s	–	–	0	1.17	–	–	0	74.38
*a*·10^2^	61.29	24.30	61.51	30.06	70.45	59.59	67.01	67.35
*n*·10^3^	0	0	0.99	27.61	21.28	277.39	0	0
$R_{\mathrm{adj}}^{2}$	0.9989	0.9999	0.9988	0.9998	0.9987	0.9999	0.9989	0.9999
AIC	74.0	58.6	75.9	64.6	74.3	57.0	71.4	56.7
MSC	6.4	8.5	6.1	7.9	6.1	8.6	6.4	8.5

## Data Availability

The original contributions presented in this study are included in the article. Further inquiries can be directed to the corresponding author.
